# Supplementary material for: Learning acquisition of consistent leader–follower relationships depends on implicit haptic interactions
Source: Sci Rep. 2023 Mar 1;13:3476. doi: 10.1038/s41598-023-29722-6 (PMC9977766; doi:10.1038/s41598-023-29722-6)
Supplement: Supplementary file 1 — Supplementary Information. [file 41598_2023_29722_MOESM1_ESM.docx]

**Supplementary Information for:**

**Learning acquisition of consistent leader-follower relationships depends on implicit haptic interactions**

Asuka Takai, Qiushi Fu, Yuzuru Doibata, Giuseppe Lisi, Toshiki Tsuchiya,

Keivan Mojtahedi, Toshinori Yoshioka, Mitsuo Kawato, Jun Morimoto, Marco Santello

Co-corresponding authors: Jun Morimoto, Marco Santello

Email: [xmorimo@atr.jp](mailto:xmorimo@atr.jp); [marco.santello@asu.edu](mailto:marco.santello@asu.edu)

**This PDF file includes:**

Supplementary text

Figures S1 to S4

***Supplementary Results***

***1. Simulation of feasible space of dyadic motor interactions****.* Computer simulations using minimum jerk hand kinematics (**Supplementary** **Figure S1a**) suggested that the range of mean hand position difference (Left – Right) compatible with the task’s spatial and temporal requirements is -0.02 m to 0.06 m (**Supplementary** **Figure S1b**). The beam angle during these coordinated movement is shown in **Supplementary** **Figure S1c**. Note that some coordination strategies can lead to a smaller peak beam angle, thus denoting a potential optimization gradient.

*
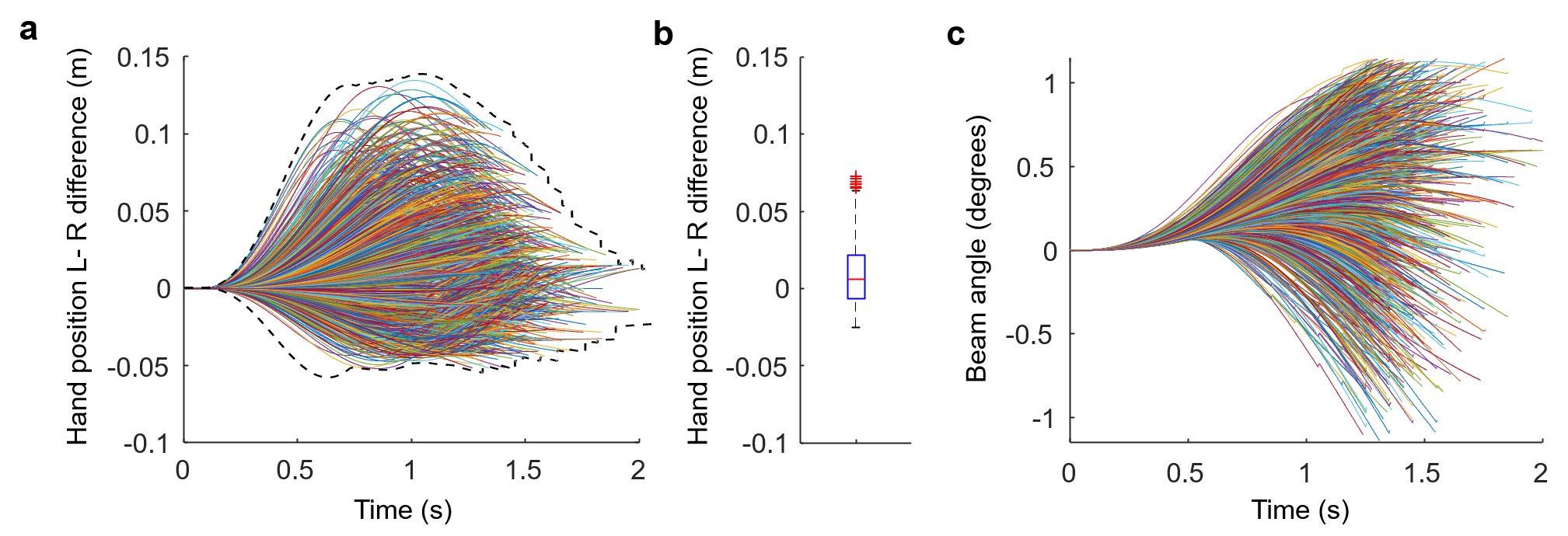
*

**Supplementary** **Figure S1. Simulation of feasible coordination strategies.** **a** Hand position differences (Left – Right) used in successful trial simulations (*n* = 1500) and the boundaries of hand position strategies. **b** Distribution of mean hand position differences (Left – Right) of feasible coordination strategies. Vertical bars denote 1.5 interquartile range. **c** Beam angle during successful trials (*n* = 1500). Each colored line in **a** and **c** denote one successful trial simulation.

***2. Total number of trials and movement speed across stiffness conditions****.* Participants were able to accomplish 40 successful trials across all stiffness conditions with only a few failed trials (significant effect of Condition, Kruskal-Wallis test, χ^2^ = 8.2, *p* = 0.016; **Supplementary** **Figure S2a**). Movement speed significantly increased across successful trials in all conditions (two-way mixed ANOVA, significant main effect of Trial on movement speed: F(1,21) = 21.54, *p* < 0.001, **Supplementary** **Figure S2b**).


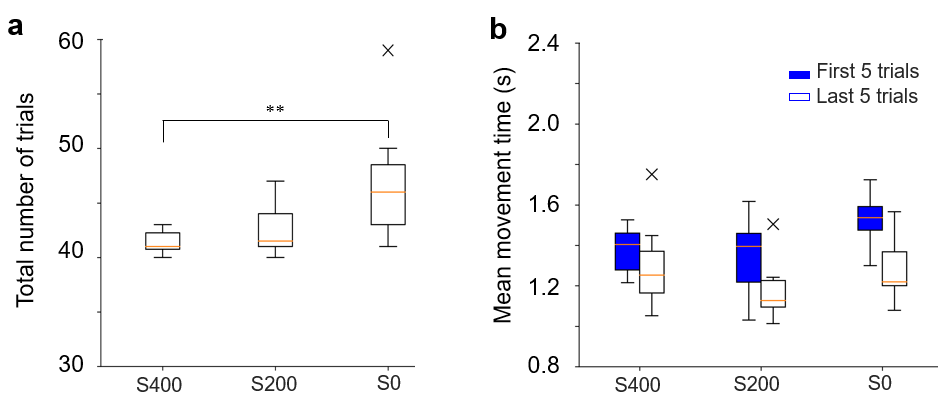


**Supplementary** **Figure S2. a** Total number of trials required to accomplish 40 successful trials across stiffness conditions. Asterisks denote statistically significant differences between conditions (** denote *p* < 0.01). **b** Comparison of movement time between the first and last five successful trials within each stiffness condition. Data shown in each panel are from 48 subjects (24 dyads). Vertical bars denote 1.5 interquartile range. The ‘🞨’ symbols in the whisker plot denote outliers beyond the whiskers.

***3. Dyadic strategy optimization***. Simulation of the effects of feasible dyadic interactions on maximum beam angle showed that the optimization landscape is approximately ‘<’ shaped with a global minimum (**Supplementary** **Figure S3a**). The experimental results show that only the S400 dyads could learn to systematically operate in this space (blue crosses, **Supplementary** **Figure S3b**). A ‘<’ shaped data distribution indicates that the optimal strategy to minimize maximum beam angle is approximately 0.02 m Left-Right handle position difference. If the left handle lead is too large or too small, the beam angle would be greater.

**
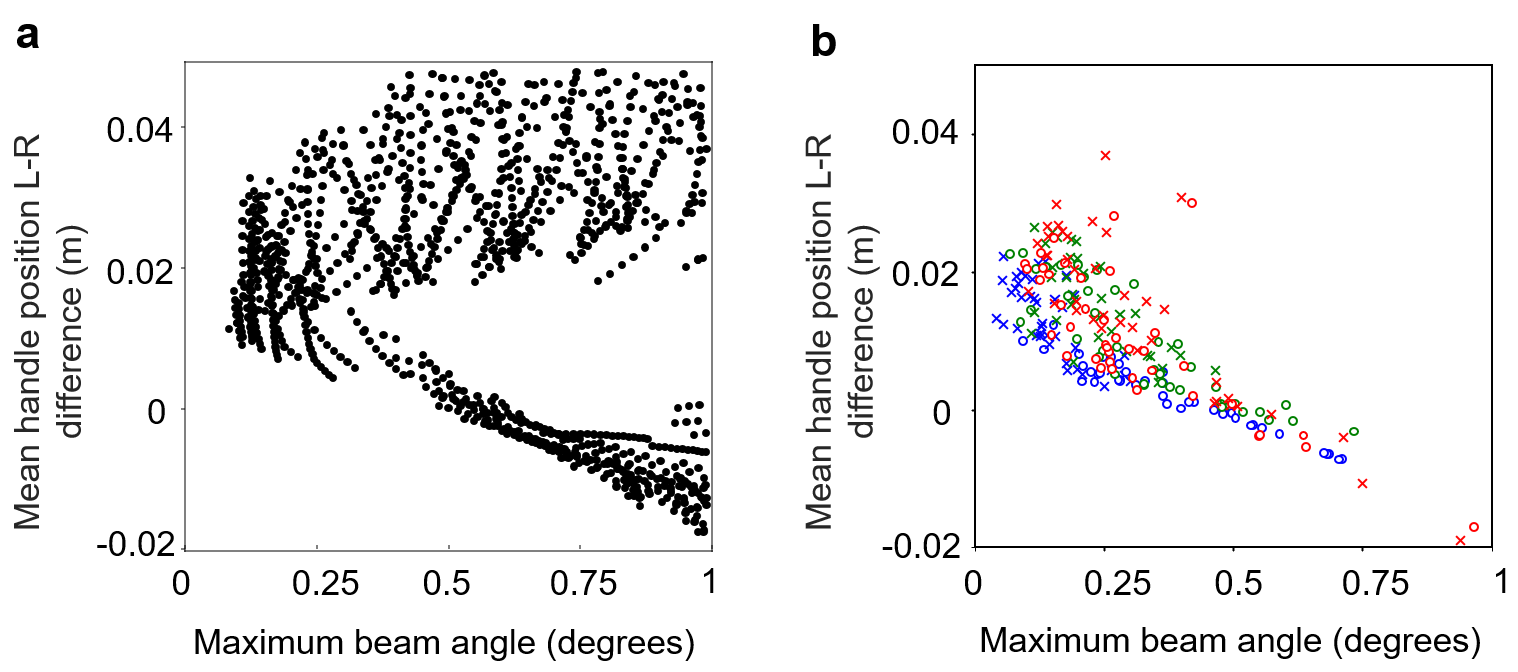
**

**Supplementary** **Figure S3. Motor coordination optimization.** **a** Simulated results (sampled from 1500 successful simulations) of successful relations between mean hand position difference and maximum beam angle. **b** Results from first and last five successful trials (circles and crosses, respectively) from all dyads. Blue, green, and red symbols denote data from individual dyads in the S400, S200 and S0 conditions, respectively.

***4. Representative plots from the S200 condition.***

***
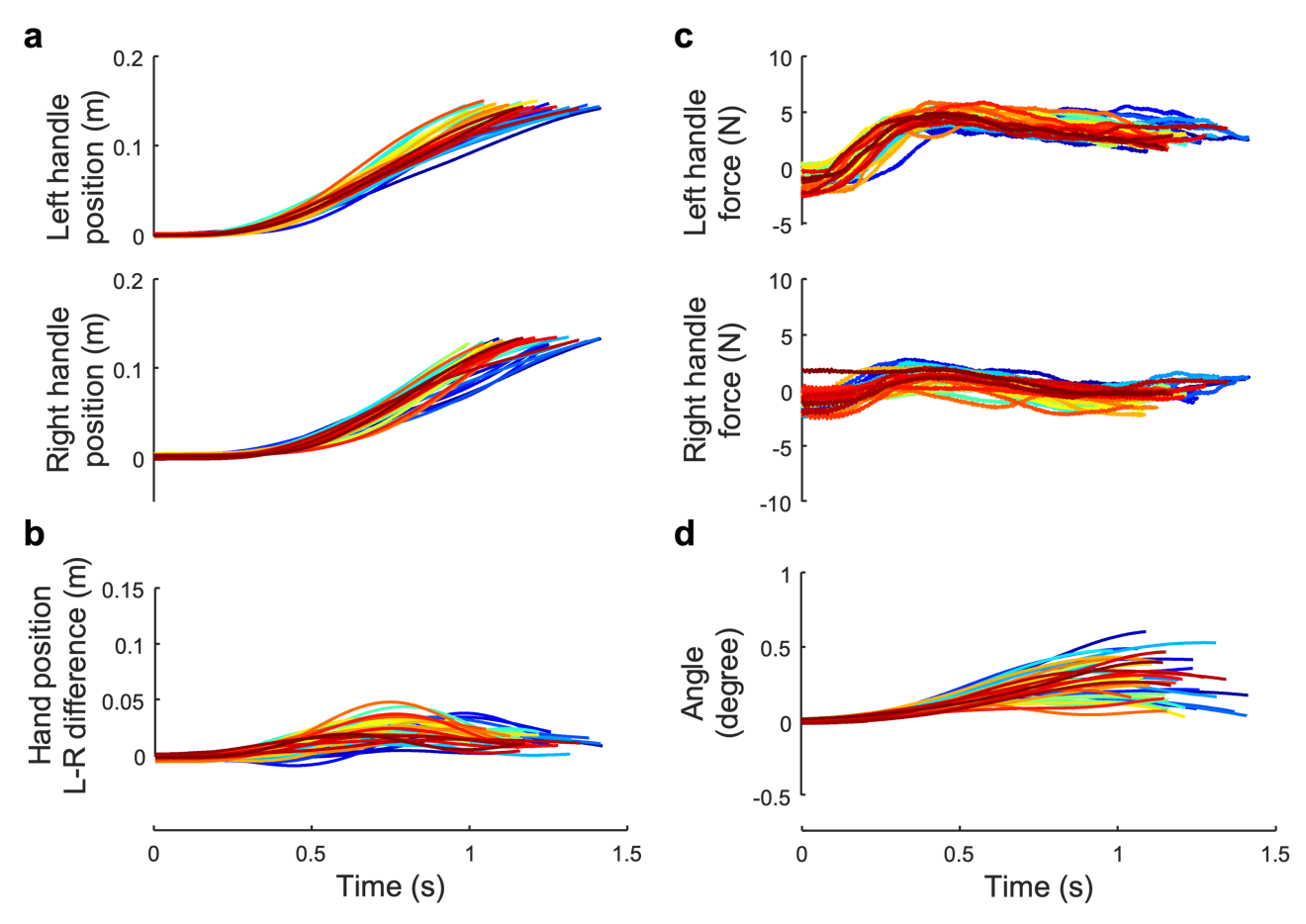
***

**Supplementary Figure S4. Changes in motor behavior of paired individuals across successful trials (S200 condition).** Early trials from the S200 condition are plotted as blue traces, and subsequent trials are denoted by ’warmer’ colors. **a** Left and right handle movements performed by a representative dyad. **b** Hand position difference from the same dyad shown in **a**. **c** Measured left and right handle force from the same dyad shown in **a**. **d** Maximum beam angle from individual successful trials from the same dyad shown in **a**. Mixed rainbow-like color distributions indicate weak changes of behavior across trials. Each plot shows data from 40 successful trials.
